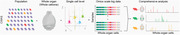# Whole‐brain Cellome‐Wide Association Study (CWAS) Unveils Ultra‐Early Neurodegeneration Phenomena in a 3D Spatial Cell Atlas

**DOI:** 10.1002/alz.093774

**Published:** 2025-01-09

**Authors:** Tomoki T Mitani, Kosei Yamaura, Katsuhiko Matsumoto, Rikuhiro Yamada, Etsuo A Susaki, Hiroki R Ueda

**Affiliations:** ^1^ RIKEN Center for Biosystems Dynamics Research, Suita‐shi, Osaka Japan

## Abstract

**Background:**

In aging societies, neurodegenerative diseases, such as Alzheimer's disease, are receiving attention. These diseases are primary targets for preemptive medicine, emphasizing the importance of early detection and preventive treatment before the onset of severe, treatment‐resistant damages. However, there is a lack of comprehensive investigation of lesions and molecular targets in the entire organ, whereas spatial identification of early‐stage lesions is potentially overlooked at the single‐cell level.

**Method:**

Here, we propose a novel approach, CWAS (whole body/organ cellome‐wide association studies), which integrates and analyzes spatio‐temporal cellular information across the entire mouse organ into a comprehensive organ cell atlas using tissue‐clearing imaging technologies [1,2]. [1] Murakami TC, et al. Nat Neurosci. 2018;21(4):625‐637. [2] Matsumoto K*, Mitani TT*, Horiguchi SA, et al*. (*equal contribution) Nat Protoc. 2019;14(12):3506‐3537.

**Result:**

In our initial application to neurodegenerative models, we have been able to identify previously unreported neural degenerations at specific times and locations at the single‐cell level. In addition, our time‐series analysis in a mouse model of Alzheimer's disease revealed the possible simultaneous onset of amyloid deposition and neurodegeneration, challenging the traditional amyloid hypothesis. Furthermore, we were able to identify unique multicellular inflammation networks surrounding neurodegenerative lesions in several disease models.

**Conclusion:**

Our findings establish the concept of "spatial cellomics" realized through spatially precise organ mapping and integrated multiomics analysis at the single‐cell level. This approach shifts pathology, providing deeper insight into disease mechanisms and preventive strategies.